# Quaternary taphonomy: understanding the past through traces

**DOI:** 10.1038/s41598-022-10473-9

**Published:** 2022-05-04

**Authors:** Ruth Blasco

**Affiliations:** 1 Institut Català de Paleoecologia Humana i Evolució Social (IPHES-CERCA), Zona Educacional 4, Campus Sescelades URV (Edifici W3), 43007 Tarragona, Spain; 2 Departament d’Història i Història de l’Art, Universitat Rovira i Virgili, Avinguda de Catalunya 35, 43002 Tarragona, Spain

**Keywords:** Palaeontology, Geology

## Abstract

Taphonomical analysis allows us to understand the processes that underlie site formation, as well as provide insights into the modification and composition of studied fossil materials. Taphonomy has become crucial to many scientific fields, providing conceptual advances through a renewal of models, protocols, and paradigms. In these studies, trans-disciplinary approaches (geology, palaeontology, biology, ecology, archaeology) have been developed using a wide array of methodologies. In addition, experimental work on modern assemblages, focusing on specific geological and biological processes (‘actualism’), are used to make referential data and proxies. This Collection contributes to the field’s methodological development, while gathering research articles investigating Quaternary period bone assemblages, with special interest in the Pleistocene.

Fossil vertebrate assemblages from archaeological sites pose an interpretive challenge for modern-day researchers. Humans, carnivores, rodents, water courses, geochemical solutions, and rock-falls are just some of the agents and processes that could produce or alter an osteological accumulation. Discriminating between products derived from human behaviour, and those produced by physical, chemical, geological, or biological processes, is the first step in tackling any archaeological study. Taphonomy, a discipline that addresses the origin and history of accumulations from the perspective of site formation, provides an essential multi- and trans-disciplinary framework for making accurate interpretations^1^.

The definition of Taphonomy was first established by Efremov^2^ as the study of living organisms’ transition from the biosphere to the lithosphere—that is, the study of the processes affecting the transition of a past living organisms’ remains (and their signatures) to the lithosphere, as can be observed in prehistoric sites. However, this conceptual subsystem of Palaeontology is not only applicable to prehistoric times, and it may cover a broader time range when more recent historical accounts are examined (e.g.^3–5^). Taphonomy, however, did not really emerge as a scientific discipline within Archaeology until the 1980s, when studies took a new direction, especially those linked to the Plio-Pleistocene record^6–8^. The focus of taphonomic research was then placed on comprehending how palaeoecological and palaeobiological information had been altered during this transition, establishing links between taphocoenoses and palaeobiocoenoses^9,10^.

The identification of bone surface modifications generated by different taphonomic agents is a key element in modern archaeology and applied forensic sciences. Amongst the different types of bone damage, gnawing marks contain valuable information to explore and understand the formation processes of archeo-paleontological sites, environments, and their associated ecosystems. Many authors have paid special interest to tooth marks generated by different animals, either through the consumption of soft tissues (e.g., meat, tendon, cartilage) or other practices of biological origin (e.g.^11^). This Collection has several articles focused on the taphonomic signature that carnivores such as felids, canids, hyenas, and ursids leave on bones^12–16^. Of special interest is the study conducted by Courtenay et al.^15^, in which a new alternative approach using 3D modelling, geometric morphometrics, and machine learning algorithms is proposed to differentiate the tooth marks of different predators; their approach reaches a classification rate of between 88 and 98% accuracy, with balanced overall evaluation metrics across all datasets. This study, together with those developed by Yravedra et al.^12^, Cifuentes-Alcobendas and Domínguez-Rodrigo^17^, and Domínguez-Rodrigo et al.^18^ in this Collection, demonstrates that these advanced data science techniques, combined with other taphonomic evidence sources, provide a valuable contribution to the identification of agents in archaeological sites. The application of new technology in Taphonomy has undoubtedly led to exponential growth in the field, through the incorporation, updating, and continuous evaluation of new methodological approaches. In this regard, the study by Domínguez-Rodrigo et al.^18^ is relevant; by using artificial intelligence through computer vision techniques—based on convolutional neural networks—the authors report the highest documented rate to date of accurate classification (92%) of cut, tooth, and trampling marks in controlled experimental bone modifications.

Quaternary taphonomic studies provide knowledge of fossil humans, their ecologies, and their cultures. For this reason, this Collection treats as its nucleus those studies focusing on subsistence strategies and hominin behaviour, with a special emphasis on the Pleistocene (although studies framed in the Holocene are also included; e.g.^19–27^). These studies are essential for understanding the origin and history of prehistoric sites, as well as the climatic and environmental contexts in which humans lived and evolved. Amongst these contributions, I would like to highlight the work of Daujeard et al.^20^, which presents some of the earliest evidence of cut, percussion, and human gnawing marks on faunal specimens associated with lithic knapping tasks in a well-documented stratified cave context in North Africa (Grotte des Rhinocéros at Casablanca, Morocco). As an example of multidisciplinarity applied to subsistence and behavioural studies, it is worth highlighting the work of Balzeau et al.^26^, which includes geochronological data (^14^C and OSL), Zooarchaeology by Mass Spectrometry (ZooMS) and ancient DNA data, geological and stratigraphic information, and taphonomic analysis of the archaeological context and Neanderthal skeleton of the La Ferrassie 8 (LF8), in order to evaluate if a burial is the most parsimonious explanation for LF8.

The Collection also includes experimental works on modern assemblages with the aim of creating models and analogies that help us infer the processes that occurred in the past and understand the formation processes of bone accumulations at archaeological sites. An example is the work developed by Duches et al.^28^, where the authors conduct ballistic experiments to distinguish projectile impact marks from other taphonomic modifications, with the objective of developing a widely applicable diagnostic framework to an archaeological context dated to the Late Glacial in the Italian Alps (Riparo I of Grotte Verdi di Pradis).

Finally, the present Collection is still open for submissions on a rolling basis, and with new studies continuing to be submitted, we expect the Collection to serve as a one-stop overview of current research in Taphonomy.
